# Assessing the presence of selection bias in meta-analyses of randomised trials using baseline heterogeneity

**DOI:** 10.1186/1745-6215-14-S1-O96

**Published:** 2013-11-29

**Authors:** Laura Clark, Caroline Fairhurst, Catherine Hewitt, Yvonne Birks, Sally Brabyn, Sarah Cockayne, Sara Rodgers, Katherine Hicks, Robert Hodgson, Elizabeth Littlewood, David Torgerson

**Affiliations:** 1 University of York, York, UK

## Objective

To assess the likelihood of biased trials contained in 12 recently published meta-analyses.

## Design

A review of component randomised controlled trials of systematic reviews with 12 meta analyses.

## Data sources

12 recently published systematic reviews with 503 component randomised trials, which were published in the BMJ, Lancet, JAMA and The Annals of Internal Medicine up until May 2012.

## Eligibility criteria for selecting studies

Systematic reviews were eligible for inclusion if they included only randomised controlled trials (RCTs). We obtained the full text for the component RCTs of the 12 systematic reviews. We excluded RCTs which were not available in English from our review and analysis.

## Results

Five of the 12 meta-analyses exhibited heterogeneity (I^2^ >0.30) in age differences, when there should have been none, with two having significant or substantial heterogeneity (I^2^ > 0.50). In three meta-analyses we found that the age of intervention group was statistically significantly higher than in the control group. Two meta-analysis had a distribution of p-values that were inconsistent with chance. Meta-regression explained some of the observed heterogeneity in two meta-analyses as a consequence of poor allocation concealment.

## Conclusions

The majority of a sample of recent meta-analyses showed that there were signs of imbalance and or heterogeneity in ages between treatment groups, when there should have been none. Systematic reviewers might consider using the techniques described here to assess the validity of their findings.

